# The effects of anxiety and external attentional focus on postural control in patients with Parkinson's disease

**DOI:** 10.1371/journal.pone.0192168

**Published:** 2018-02-01

**Authors:** Seyede Zohreh Jazaeri, Akram Azad, Hajar Mehdizadeh, Seyed Amirhassan Habibi, Mahbubeh Mandehgary Najafabadi, Zakieh Sadat Saberi, Hawre Rahimzadegan, Saeed Moradi, Saeed Behzadipour, Mohamad Parnianpour, Ghorban Taghizadeh, Kinda Khalaf

**Affiliations:** 1 Department of Occupational Therapy, School of Rehabilitation Sciences, Iran University of Medical Sciences, Tehran, Iran; 2 Department of Neuroscience, School of Advanced Technologies in Medicine, Tehran University of Medical Sciences, Tehran, Iran; 3 Department of Occupational Therapy, School of Rehabilitation, Tehran University of Medical Sciences, Tehran, Iran; 4 Department of Neurology, Movement Disorder Clinic, Rasool Akram Hospital, Iran University of Medical Sciences, Tehran, Iran; 5 Rasool Akram Hospital, Iran University of Medical Sciences, Tehran, Iran; 6 Department of Mechanical Engineering, Sharif University of Technology, Tehran, Iran; 7 Mowafaghian Research Center in Neurorehabilitation Technologies, Tehran, Iran; 8 Rehabilitation Research Center, School of Rehabilitation Sciences, Iran University of Medical Sciences, Tehran, Iran; 9 Department of Biomedical Engineering, Khalifa University of Science, Technology and Research, Abu Dhabi, UAE; Philadelphia VA Medical Center, UNITED STATES

## Abstract

**Background:**

Although anxiety is a common non-motor outcome of Parkinson's disease (PD) affecting 40% of patients, little attention has been paid so far to its effects on balance impairment and postural control. Improvement of postural control through focusing on the environment (i.e. external focus) has been reported, but the role of anxiety, as a confounding variable, remains unclear.

**Objectives:**

This study aimed to investigate the influence of anxiety and attentional focus instruction on the standing postural control of PD patients.

**Methods:**

Thirty-four patients with PD (17 with high anxiety (HA-PD) and 17 with low anxiety (LA-PD)), as well as 17 gender- and age-matched healthy control subjects (HC) participated in the study. Postural control was evaluated using a combination of two levels of postural difficulty (standing on a rigid force plate surface with open eyes (RO) and standing on a foam surface with open eyes (FO)), as well as three attentional focus instructions (internal, external and no focus).

**Results:**

Only the HA-PD group demonstrated significant postural control impairment as compared to the control, as indicated by significantly greater postural sway measures. Moreover, external focus significantly reduced postural sway in all participants especially during the FO condition.

**Conclusion:**

The results of the current study provide evidence that anxiety influences balance control and postural stability in patients with PD, particularly those with high levels of anxiety. The results also confirmed that external focus is a potential strategy that significantly improves the postural control of these patients. Further investigation of clinical applicability is warranted towards developing effective therapeutic and rehabilitative treatment plans.

## Introduction

Impaired postural control is a common symptom in patients with Parkinson's disease (PD), where the severity is known to increase along with the disease progression, predisposing the patients to balance problems, unexpected falls, and various types of injuries [1, 2]. Previous studies have reported conflicting results on the postural control of PD patients. For example, while some studies found greater postural sway in PD patients, as compared with age-matched healthy counterparts [3], others reported decreased postural sway [4], or no difference, as compared to the control group [5]. Recent studies suggest that common medical interventions (including dopaminergic drugs and deep brain stimulation) may not be effective for sufficiently restoring the postural control of PD patients [6]. From a clinical perspective, further investigation of postural control characteristics in these patients is needed towards developing effective therapeutic and rehabilitative plans and strategies.

Anxiety is a prevalent non-motor symptom of PD, affecting up to 40% of patients [7]. Previously considered a consequence of the disease and associated motor impairment, the medical community now believes that anxiety is an integral part of PD that sometimes even precedes the diagnosis or the presence of movement disorder symptoms by a few years. While the precise pathophysiology of anxiety in PD patients remains elusive, it has been suggested that it may be a pathological reaction to other PD symptoms, such as motor impairment [8]. It has also been attributed to more direct physiological causes, such as decreased dopaminergic transmission of the basal ganglia [9]. Recent functional magnetic resonance imaging (fMRI) studies of PD patients confirm that impaired dopaminergic input to the amygdala and limbic system are indeed associated with emotional abnormalities [10]. These studies also demonstrate the neural links between the regions in the brain controlling emotions and those responsible for balance control. Specifically, the amygdala and limbic structures, which play an important role in the processing of emotions such as anxiety, are linked via efferent projections to the brain regions involved in postural control, including the basal ganglia, nucleus accumbans, reticular formation and vestibular nuclei [11]. Up to date, few studies have investigated the effect of anxiety on the balance and postural control of PD patients. In their recent study, Ehgoetz Martens et al. (2017) evaluated the influence of anxiety on the balance of PD patients using a virtual environment. They reported high anxiety levels, particularly under high threat (elevated plank) conditions [12]. On the other hand, since a high correlation was found between depression, anxiety and cognitive scores, the effect of anxiety was not conclusively independent. To the best of our knowledge, no studies thus far have explored the influence of anxiety on the postural control of PD patients in real-life conditions with different levels of postural difficulty while considering cognitive scores.

It has been shown that the basal ganglia play an important role in the “automaticity” of movement, resulting in relatively effortless control of well-learned and coordinated movements (i.e. controlling movement with no or little attentional demand) [13, 14]. Degeneration of the basal ganglia decreases the automaticity of movements in PD patients, and therefore, they tend to consciously control the movement in order to compensate for the decreased automaticity [15]. This results in increased attentional demand of movement control in these patients [16]. Previous studies have suggested that attentional focus affects motor performance including balance [17, 18]. Attentional focus in this regard is defined as a location to which an individual directs his/her attention while performing a particular motor task. For example, directing one’s attention towards the body is referred to as “internal focus”, while directing it towards an external environment is labeled as “external focus” [17, 18]. Based on the “constrained attention hypothesis”, internal focus interferes with automatic motor control processing, resulting in decreased neuromuscular degrees of freedom. Conversely, external focus stimulates automatic motor control, which results in fast reaction to postural perturbations; hence potentially leading to increased postural stability [18, 19].

Indeed, it has been previously shown that external attentional focus results in decreased postural sway (i.e. increased postural stability/control) of patients with PD [14, 17, 20]. Wulf et al. (2009) used an inflated rubber disk placed on a force platform for evaluating the effects of attentional focus on the standing postural control of patients with PD during open eyes conditions. The participants were asked to focus on reducing the movement of their feet as well as the disk movements during internal and external focus condition, respectively, while they were required to stand still during the condition of no focus [14]. Beck et al. (2016) conducted the same protocol in PD participants, but they used a moving force platform instead of the inflated disk [20]. In addition to using moving platform during open eyes condition, Landers et al. (2005) considered both open and closed eyes conditions using a static force platform and two rectangular pieces of paper placed as focus cues during the external focus condition. The participants were asked to concentrate their focus on their feet and the rectangular papers under their feet during internal and external focus conditions, respectively [17]. The condition of no focus instruction was similar to Wulf et al. In all these studies, the participants were required to look forward and concentrate on the internal or external cues without looking at them. Landers et al. found external focus advantageous only in the case of the moving force platform (which induces a combination of proprioceptive and vestibular challenge), as compared to the open-eyes condition, but not during the closed-eyes condition. Conversely, Wulf et al. (2007) showed increased effects of the external focus on postural control while standing on a foam surface (i.e. proprioceptive challenge condition), as compared to standing on a rigid surface in young healthy adults [21], possibly due to the fact that standing postural control mainly depends on somatosensory with proprioception [22]. Despite the multiple studies that have confirmed that external attentional focus results in decreased postural sway (increased postural stability/control) of patients with PD, the impact of introducing a proprioceptive challenge remains largely elusive. Furthermore, none of the aforementioned studies included gender and age-matched healthy subjects. No clear quantitative evidence was provided on attentional focus instruction as related to the improvement of postural stability in anxious PD patients.

Therefore, the aim of this study was twofold: 1. To investigate the influence of anxiety on postural stability in PD patients under different postural difficulty levels, and 2. To study the effects of different attentional focus instructions on anxiety-related postural control of these patients in different levels of postural difficulty as compared with gender- and age-matched healthy counterparts.

## Materials and methods

### Participants

Thirty-four subjects with idiopathic PD (neurologist-diagnosed using UK brain Bank criteria for the diagnosis of idiopathic PD), and 17 gender- and age-matched healthy control subjects (HC) participated in this study. All participants completed the hospital anxiety and depression scale (HADS), as well as the mini mental state examination (MMSE). PD participants were included if they achieved Hoehn and Yahr scores of I-III, MMSE scores ≥ 24, and were able to walk without any assistive devices for a distance of at least 10 meters. Further inclusion criteria involved being free from any neurological disorders (other than PD), dyskinesia, dizziness, dementia, diabetes, vestibular dysfunction, orthopedic disorders, history of falling within the past year, surgical intervention for PD, as well as depression (i.e., scores equal or less than 7 on the depression subscale of HADS). Healthy subjects were included if they did not have anxiety (i.e. scores equal or less than 7 on the anxiety subscale of HADS), depression (i.e. scores equal or less than 7 on the depression subscale of HADS), neurologic or orthopedic disorders, vestibular dysfunction and history of falling within the past year. Both PD and healthy participants were excluded if they experienced falling during the test. Because of recent evidence about the importance of testing balance using a wide spectrum of anxious PD participants [12, 23], we divided the PD participants into low-anxiety (LA-PD) and high-anxiety (HA-PD) groups according to their anxiety score on the anxiety subscale of HADS. PD participants who obtained scores of less than 11 were grouped as LA-PD, while those whose scores were equal or greater than 11 were grouped as HA-PD [24]. Based on literature, HADS is considered a valid, consistent, precise and responsive scale for use in the anxiety/depression assessment of PD patients [25]. Since recent studies have shown that dopaminergic treatment does not significantly improve the postural control of PD participants [26, 27], all PD participants were assessed during the “Off”-drug phase (i.e. after at least 12 h withdrawal from regular dopaminergic medication [23]). All other assessments were also performed during the “Off”-drug phase. Unified Parkinson's Disease Rating Scale motor section (URPDS III) was used to evaluate the motor symptoms of the patients. In addition, all participants completed the clock drawing test for assessing cognitive function [28], Beck Depression Inventory for evaluating depression [29], and fatigue severity scale for assessing fatigue [30]. Pain was also evaluated using a visual analogue scale. The study was approved by the Ethics Committee of Iran University of Medical Sciences. Before the beginning of testing, all participants carefully read and signed an informed consent form.

### Procedure

The center of pressure (COP) sway, a quantitative indicator of postural control, was measured using a Kistler force plate as the participants stood barefoot in a bipedal stance position with their arms relaxed at their sides. COP data were collected at a sampling frequency of 100 Hz. Postural control was assessed under combined conditions consisting of two levels of postural difficulty (standing on the rigid surface of the force plate with open eyes (RO), and standing on a 10.5 cm thick foam surface with open eyes (FO)), as well as three different attentional focus instructions (external, internal, and no focus instruction). During the external focus condition, the participants were asked to focus on rectangular papers (30.5 × 17 cm, one under each foot) which were placed on the force plate or foam without looking at them. During the internal focus condition, the participants were required to concentrate on their feet without looking at them. During the no focus condition, the participants were instructed to stand still. Under all experimental conditions, the participants were asked to look straight ahead. The participants performed two 70-second trials separated by a 60 second rest interval. Five minutes rest intervals were adopted between experimental conditions. Different experimental conditions were performed randomly for each subject. None of the participants reported pain at any point during the experiment.

### Data analysis

Anterior-posterior (AP) and medial-lateral (ML) displacements of the COP were measured. In order to ensure evaluating both static and dynamic aspects of postural control performance, postural sway measures with high reliability [26], including the mean velocity, the standard deviation (SD) of velocity along both AP and ML directions, the total phase plane portrait and the path length were calculated based on the COP time series. We hypothesized, based on literature, that postural instability (i.e. impaired postural control) leads to increased postural sway.

### Statistical analysis

Assessing normal distribution of the data using the Shapiro-Wilk test showed that all postural sway measures were randomly distributed. An average value for each postural sway measure was calculated over two trials of the same condition. The main and interaction effects of postural difficulty and focus conditions on the postural sway measures in the LA-PD, HA-PD and HC groups were analyzed using a 3 × 3 × 2 (focus condition × group × postural difficulty) three-way repeated measure analysis of variance (ANOVA). Post hoc analyses using the Bonferroni adjustment method were performed to evaluate the effect of each focus condition (no focus instruction, internal focus and external focus) and groups (control, LA-PD and HA-PD) on postural sway measures during standing on rigid and foam surface conditions. The *α* level was set to 0.05.

## Results

Table 1 depicts the demographic and clinical characteristics of participants. No significant difference was found between the groups with the exception of their scores on the Anxiety subscale of HADS (F _(2, 48)_ = 140.86, P = 0.000). The post hoc analysis of the Anxiety subscale of HADS showed that the anxiety was significantly greater in the HA-PD group as compared to the control and LA-PD groups. No significant correlation was found between anxiety (anxiety subscale of HADS) and cognitive function (r = -.024, P = 0.09 for MMSE; r = 0.21, P = 0.15 for clock drawing test). Descriptive data of the postural sway measures in different conditions of postural difficulty and focus are presented in Table 2. Table 3 presents a summary of the ANOVA results for the postural sway measures.

**Table 1:** Demographic and clinical characteristics of participants.

	Healthy	LA-PD^a^	HA-PD^b^	P-value
**N**	17	17	17	-
**Sex**	13 Male, 4 female	13 Male, 4 female	13 Male, 4 female	-
**Age (years)**	64.18 ± 7.33	61.94 ± 8.40	63.06 ± 11.62	0.78
**Height (cm)**	167.44 ± 7.45	165.47 ± 9.35	168.26 ± 8.71	0.60
**Weight (kg)**	73.32 ± 12.95	69.30 ± 11.26	68.76 ± 14.70	0.54
**MMSE**^c^	28.18 ± 1.74	27.76 ± 1.99	26.94 ± 1.95	0.17
**Clock drawing test**	1.12 ± 0.33	1.29 ± 0.47	1.41 ± 0.51	0.16
**Hoehn & Yahr Scale**	-	2 ± 0.61	2.41 ± 0.71	0.08
**UPDRS-III**^d^	-	22.02 ± 8.94	25.41 ± 8.13	0.25
**Beck Depression Inventory**	3.12 ± 1.32	3.59 ± 1.18	3.29 ± 1.05	0.51
**Depression subscale of HADS**^e^	3.65 ± 2.37	4.76 ± 1.75	5.06 ± 2.25	0.14
**Anxiety subscale of HADS**	3.53 ± 1.97	5.41 ± 2.83	16.41 ± 2.37*	***0*.*000***
**Fatigue severity scale**	2.47 ± 0.80	2.94 ± 0.90	2.71 ± 0.85	0.28

Note

^a^LA-PD, PD participants with low anxiety

^b^HA-PD, PD participants with high anxiety

^c^ MMSE, Mini Mental State Examination

^d^UPDRS-III, Unified Parkinson's Disease Rating Scale motor subsection

^e^HADS, Hospital Anxiety and Depression Scale

*shows the group significantly differ from the other two groups (indicated by Bonferroni post-hoc analysis)

**Table 2:** Descriptive data for postural sway measures in different postural difficulty and focus conditions.

Focus condition	No focus instruction			Internal focus			External focus		
Group	Healthy	LA-PD^a^	HA-PD^b^	Healthy	LA-PD	HA-PD	Healthy	LA-PD	HA-PD
**Standing on rigid surface with open eyes**									
Mean velocity (cm/s)	5.16±0.51	5.38±1.39	5.82±1.34	5.20±0.45	5.46±1.42	5.81±1.36	5.07±0.52	5.24±1.34	5.70±1.34
SD of velocity (M.L^c^) (cm/s)	3.67±0.44	3.83±1.16	4.09±1.01	3.60±0.34	3.90±1.26	4.12±1.13	3.64±0.38	3.73±0.98	4.07±0.96
SD of velocity (A.P^d^) (cm/s)	4.59±0.46	4.72±1.13	5.20±1.16	4.68±0.42	4.78±1.11	5.20±1.15	4.47±0.49	4.61±1.17	5.04±1.18
Total phase plane portrait (arbitrary unit)	6.08±0.63	6.15±1.58	6.84±1.50	6.12±0.52	6.25±1.58	6.87±1.55	5.90±0.64	5.97±1.51	6.64±1.52
Path length (cm)	361.25±35.43	376.53±97.36	407.13±94.04	363.67±31.68	382.20±99.36	406.99±95.08	354.82±36.17	366.46±93.52	398.91±93.52
**Standing on foam surface with open eyes**									
Mean velocity (cm/s)	6.28±0.95	7.08±2.00	7.72±2.15	6.24±1.00	7.06±1.98	7.78±2.41	5.83±0.83	6.83±2.01	7.49±2.50
SD of velocity (M.L) (cm/s)	4.40±0.89	5.05±1.51	5.44±1.65	4.38±0.82	4.91±1.40	5.60±2.03	4.21±0.60	4.80±1.42	5.40±1.75
SD of velocity (A.P) (cm/s)	5.74±0.89	6.35±1.75	6.96±1.85	5.68±1.03	6.33±1.74	7.02±2.16	5.11±0.74	6.08±1.79	6.60±2.34
Total phase plane portrait (arbitrary unit)	7.45±1.29	8.19±2.29	9.07±2.48	7.38±1.38	8.07±2.23	9.21±2.95	6.92±0.92	7.79±2.27	8.73±2.91
Path length (cm)	439.45±66.18	495.60±139.89	540.58±150.28	436.76±69.74	494.57±138.58	544.72±168.63	407.80±57.91	478.41±140.69	524.19±175.40

Note:

^a^LA-PD, PD participants with low anxiety

^b^HA-PD, PD participants with high anxiety

^c^M.L, medial-lateral

^d^A.P, anterior-posterior

**Table 3:** Summary of analysis of variance of postural sway: F ratios, P values, and effect sizes by variable.

Independent variable	Mean velocity (cm/s)			SD of velocity (M.L^a^) (cm/s)			SD of velocity (A.P^b^) (cm/s)			Total phase plane portrait (arbitrary unit)			Path length (cm)		
	F	P	Partial ƞ2	F	P	Partial ƞ2	F	P	Partial ƞ2	F	P	Partial ƞ2	F	P	Partial ƞ2
**Main effect**															
Group	3.31	***0*.*04***	0.12	3.19	***0*.*05***	0.12	3.43	***0*.*04***	0.13	3.50	***0*.*04***	0.13	3.32	***0*.*04***	0.12
Postural difficulty	34.74	***0*.*000***	0.42	28.73	***0*.*000***	0.37	36.60	***0*.*000***	0.43	34.42	***0*.*000***	0.42	34.78	***0*.*000***	0.42
Focus	24.04	***0*.*000***	0.33	4.72	***0*.*01***	0.09	26.78	***0*.*000***	0.36	19.65	***0*.*000***	0.29	23.90	***0*.*000***	0.33
**Interaction effect**															
Group × Postural difficulty	1.16	0.32	0.05	1.02	0.37	0.04	1.07	0.35	0.04	0.94	0.40	0.04	1.15	0.32	0.05
Group × Focus	0.33	0.86	0.01	0.87	0.48	0.04	0.92	0.46	0.04	0.23	0.92	0.01	0.32	0.86	0.01
Postural difficulty × Focus	4.66	***0*.*01***	0.09	0.98	0.37	0.02	9.02	***0*.*000***	0.16	3.45	***0*.*04***	0.06	4.66	***0*.*01***	0.09
Group × Postural difficulty × Focus	1.18	0.32	0.05	0.89	0.47	0.04	1.32	0.27	0.05	0.61	0.66	0.03	1.19	0.32	0.05

Note:

^a^M.L, medial-lateral

^b^A.P, anterior-posterior

**Fig 1:**
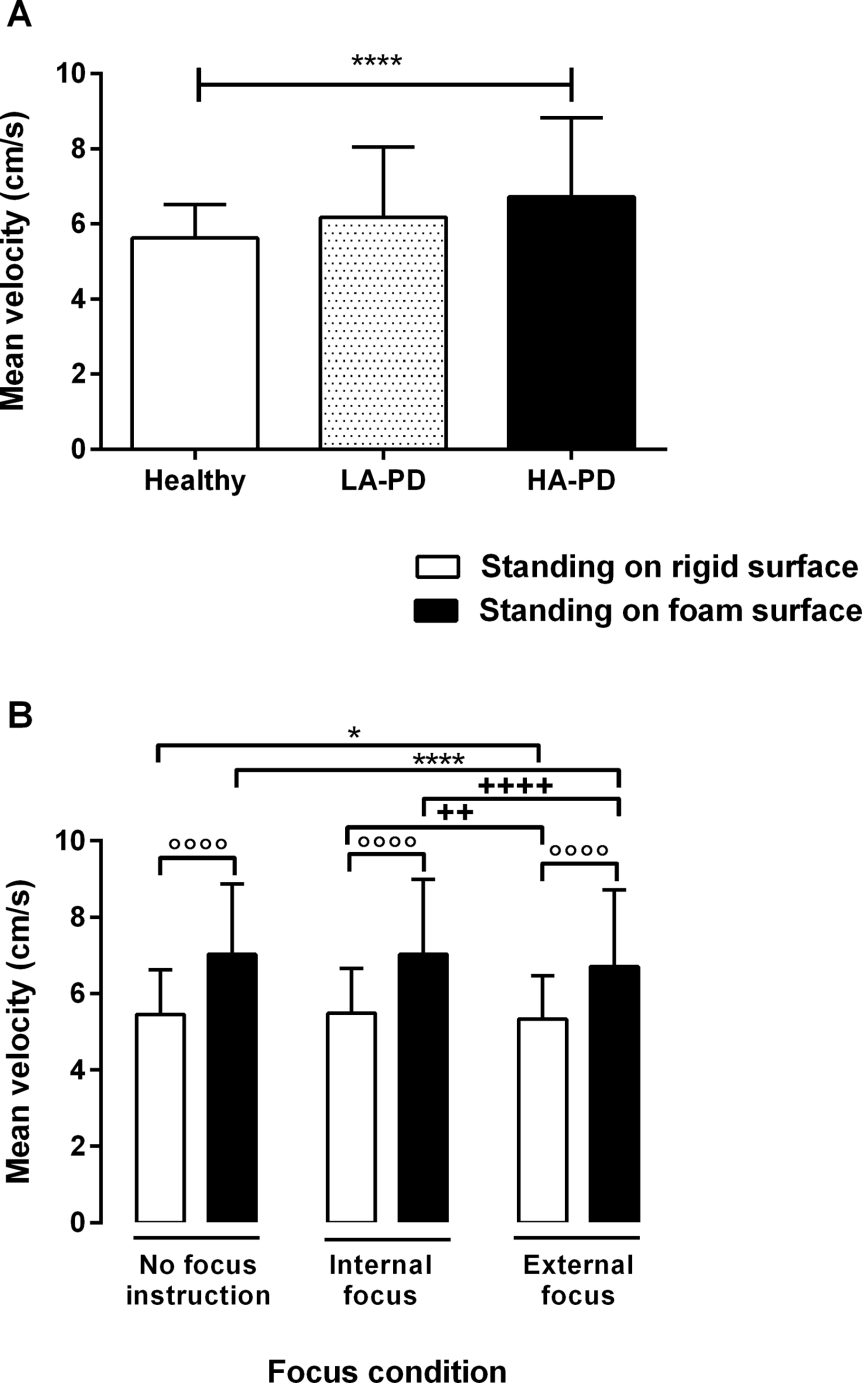
**(A) The main effect of group on mean velocity of COP**. Post hoc analysis showed that only PD participants who had a high level of anxiety showed greater mean velocity of the COP as compared to the control group (****P< 0.0001). LA-PD: PD participants with a low level of anxiety, HA-PD: PD participants with a high level of anxiety; **(B) The interaction effect of postural difficulty by focus condition on mean velocity of COP.** Post hoc analysis indicated that mean velocity of the COP was significantly greater during standing on a foam surface as compared to standing on a rigid surface (°°°°P< 0.0001). Moreover, the mean velocity of the COP decreased significantly during external focus as compared to the condition of the no focus instruction (*P<0.05 and ****P<0.0001). In addition, the mean velocity of the COP decreased significantly during the external focus as compared to internal focus condition (++P<0.01 and ++++P< 0.0001).

The results showed that all postural sway measures were significantly affected by the group, postural difficulty and focus. There were no significant interactions for the group by postural difficulty, group by focus as well as group by postural difficulty by focus. However, the results showed a significant interaction of postural difficulty by focus for the mean velocity (F _(2, 96)_ = 4.66, P = 0.01), SD of the velocity along the AP direction (F _(2, 96)_ = 9.02, P = 0.000), total phase plane portrait (F _(2, 96)_ = 3.45, P = 0.04) and path length (F _(2, 96)_ = 4.66, P = 0.01). As indicated in Fig 1 (A) using the mean velocity as an example, the Post hoc analysis showed that all postural sway measures were significantly greater in HA-PD as compared to the control group. In addition, Fig 1 (B) shows two-way interaction effects of postural difficulty by focus on the mean velocity of the COP. The results of the Post hoc analysis revealed that only the external focus of attention resulted in decreasing postural sway measures. Moreover, the effect of the external focus on decreasing the mean velocity was greater during the standing on foam condition as compared to standing on a rigid surface.

## Discussion

The purpose of this study was to investigate the postural stability of PD patients as related to their anxiety, and to explore the effects of different attentional focus instructions on these patients. The results showed that only the high anxiety group (HA-PD) demonstrated significantly greater postural sway, indicating postural instability, as compared to the control group. This result is consistent with recent studies which showed impaired standing balance under high threat conditions (for example, standing on an elevated plank placed on the ground) [12]. This may be explained based on distraction models, which argue that anxiety draws attention towards task-irrelevant stimuli, resulting in less attentional resources available for motor control processing and hence disturbed motor performance [31]. It is also possible that some of the attentional resources have already been dedicated to compensate for the sensory-motor and perceptual impairments associated with PD (e.g. changes of postural reflexes, muscular weakness, decreased anticipatory postural responses, etc.) [12]. Thus, a high level of anxiety in PD patients may overload the processing resources resulting in impaired postural control in the HA-PD group [23]. Previous studies have demonstrated changes in visual perception due to anxiety [32]. Since maintaining postural balance requires the proper integration of different sensory input, including visual, vestibular and somatosensory information [33, 34], impaired visual perception due to anxiety is hypothesized to hamper this sensory integration adding to the postural instability. Regardless of the associated mechanisms, anxiety seems to play a strong role in the postural control and balance of PD patients as confirmed by our results, where the level of anxiety is a critical component.

The current study showed that by increasing the postural difficulty level from standing on a rigid surface to standing on foam, postural sway increased in HC, LA-PD and HA-PD, indicating increased instability. Quiet upright standing is mainly dependent on proprioceptive information, where standing on a foam surface could decrease the proprioceptive information associated with the ankle joint. It has been reported that PD, as well as healthy subjects, have similar dependency on the ankle’s proprioceptive information [22]. Therefore, it is not surprising that all three groups showed increased postural sway when standing on the foam surface.

The findings of the present study confirm that external attentional focus significantly reduces the postural sway of all participants as compared to no focus instruction, and in contrast to internal attentional focus, which has no effect on the postural sway. This finding is also in alignment with previous studies, which showed the benefits of external focus as compared to no focus and internal focus instructions [14]. In the condition of external attention focus, the postural control mechanisms are allowed to regulate in an unconstrained manner (i.e. increased automaticity of postural control), resulting in more effective postural control as indicated by decreased postural sway in the current study [18, 19]. Moreover, it has been suggested that external focus results in decreased electromyographic (EMG) activity, which signifies greater motor control efficiency through recruiting motor units in a more discriminate manner and hence reducing the noise in the motor system that impedes fine motor control [19]. The specific role of decreased EMG activity in the improvement of postural stability due to external focus should be further investigated in future studies. The insignificant difference in postural sway associated with the no focus instruction was also in alignment with other studies. It may be interpreted by the fact that when individuals receive no attentional focus instruction, he/she is more likely to instinctively focus on their own movement. This may be a cautious strategy adopted by the neuromuscular system to tackle novel motor tasks, especially as related to balance [14].

An unexpected finding of the current study was the beneficial effect of external focus on postural stability, which was observed in all conditions including high anxiety (HA-PD). As previously mentioned, high levels of anxiety may significantly reduce on-task attention, resulting in a lack of sufficient attentional resources for motor control processing [31], and eventually leading to impaired postural control. High anxiety levels may also cause increased adoption of conscious motor control strategies, hence potentially leading to ineffective postural control (i.e. increased postural sway) in HA-PD [15]. On the other hand, external focus instruction attenuates anxiety through reducing vigilance of threat detection, encouraging the patient to adopt more automatic postural control [35]. It can therefore be suggested that external attention focus may potentially be a valuable strategy in the rehabilitation of PD patients towards postural control and balance improvement, including those with high anxiety levels. Further research is warranted in this regard.

Another interesting finding of this study was that external focus had a greater effect on decreasing the postural sway in more difficult postural tasks (i.e. standing on foam). Wulf et al. (2007) also reported that by increasing the level of postural difficulty (from solid surface to foam), postural sway markedly decreased with external focus [21]. Standing on a rigid surface is a relatively simple motor task, which is typically controlled rather automatically. On the other hand, standing on foam may induce more anxiety due to the disturbance in the proprioceptive information resulting from the adoption of a more conscious motor control strategy. It has also been postulated that external attention focus decreases vigilance of threat detection and hence reduces anxiety, resulting in more automatic postural control [35].

Finally, some limitations should be mentioned here. First, this study was only conducted during the Off-drug phase of patients with PD. Recent studies showed that dopaminergic treatment did not significantly improve the standing balance [26, 27]. We have not considered an additional On-drug phase in the current study for logistics purposes. Moreover, controversial results have been reported regarding the effects of dopaminergic treatment on anxiety in PD patients. Although some studies reported alleviation of anxiety during the on-drug phase [36, 37], others found no change or exacerbation of the anxiety while receiving the treatment [38, 39]. Ehgoetz Martens et al. (2017) showed that the dopaminergic medication did not significantly change the balance of LA-PD and HA-PD under high threat conditions, but slightly affected the balance of these patients under low threat conditions [12]. Future work should consider both drug phases in order to evaluate the efficacy of dopaminergic treatment in association with postural control and anxiety. Second, we did not compare patients with PD with high levels of anxiety with healthy subjects who had equally high anxiety levels. Therefore, we are unable to definitely conclude that the anxiety-induced postural instability is a specific consequence of anxiety in PD patients stemming from basal ganglia impairment. However, according to the theoretical framework, it is not expected that healthy subjects with high levels of anxiety demonstrate the same amount of postural impairments (if any) as those with PD. Healthy subjects have intact basal ganglia, and hence the competition for resources to regulate emotional and motor outcomes should be minimal. Further investigation to confirm this is recommended. Third, considering specific cognitive function (i.e. executive function) is suggested for future studies, since only global cognitive function was assessed here. Fourth, only patients with PD who had no other medical comorbidities (such as depression, neurologic or orthopedic disorders, or vestibular dysfunction) and no history of falling during the past year were included in this study. This may limit the generalizability of our results. Finally, the dominant motor phenotypes in PD, specifically, tremor-dominant and akinetic-rigid, were not considered in the current study, which is also merits further investigation.

In summary, the main contribution of this work is providing evidence that postural instability in PD patients is influenced by the presence and level of anxiety. This and the recently emerging data that anxiety is an integral part of PD that sometimes precedes the motor dysfunction, confirm that future balance studies should consider the anxiety profiles of these patients. Another important contribution is demonstrating that external attentional focus significantly improves postural control of PD patients, including those with high levels of anxiety. The clinical value of these results lies in providing a framework for accounting for anxiety in the postural and balance control of PD patients. In current clinical settings, both therapeutic treatment as well as rehabilitative interventions would benefit from decreasing the level of anxiety towards improving postural control. On the other hand, external attentional focus promises to be a potential effective strategy to improve the postural control of PD patients with various levels of anxiety.
